# Supplementary material for: Virological Passive Surveillance of Avian Influenza and Arboviruses in Wild Birds: A Two-Year Study (2023–2024) in Lombardy, Italy
Source: Microorganisms. 2025 Apr 22;13(5):958. doi: 10.3390/microorganisms13050958 (PMC12114497; doi:10.3390/microorganisms13050958)
Supplement: Supplementary file 1 [file microorganisms-13-00958-s001.zip › microorganisms-3575634-supplementary.pdf]

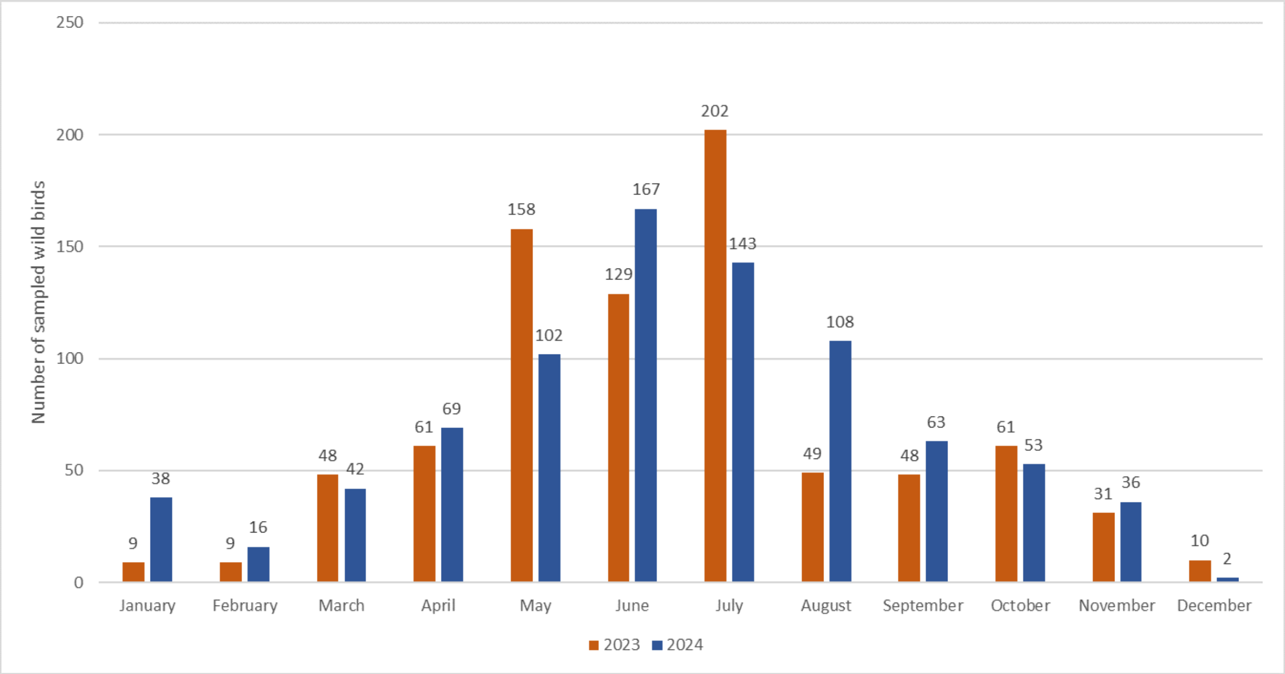

Figure S1. Monthly distribution of sampled wild birds (2023–2024). The graph shows seasonal variations in the number of deceased and sampled wild birds admitted to the Wildlife Rescue Center of Vanzago. In both 2023 and 2024, sample numbers increased in spring (March–April), peaked between May and August, and declined in autumn, reaching their lowest in winter (December–February). This trend reflects bird biology and migratory behavior, with higher admissions during the breeding, nestling and spring migration season.

Table S1. Taxonomic orders and species of wild birds analyzed during 2023–2024, with distribution by year.

Samples	Family	Species	Total	2023	2024
Accipitriformes	*Accipitridae*	Northern Goshawk *(Accipiter gentilis)*	8	7	1
		Eurasian Buzzard *(Buteo buteo)*	12	4	8
		Eurasian Sparrowhawk *(Accipiter nisus)*	5	3	2
Anseriformes	*Anatidae*	Mute Swan *(Cygnus olor)*	1	/	1
		Mallard *(Anas platyrhynchos)*	100	67	33
		Tufted Duck *(Aythya fuligula)*	1	1	/
Apodiformes	*Apodidae*	Alpine Swift *(Tachymarptis melba)*	26	9	17
		Common Swift *(Apus apus)*	107	54	53
Bucerotiformes	*Upupidae*	Eurasian Hoopoe *(Upupa epops)*	3	2	1
Caprimulgiformes	*Caprimulgidae*	European Nightjar *(Caprimulgus europaeus)*	1	1	/
Charadriiformes	*Scolopacidae*	Eurasian Woodcock *(Scolopax rusticola)*	10	4	6
	Laridae	Black-headed Gull *(Chroicocephalus ridibundus)*	17	17	/
		Yellow-legged Gull *(Larus michahellis)*	9	6	3
	Charadriidae	Northern Lapwing *(Vanellus vanellus)*	1	1	/
Ciconiiformes	*Ciconiidae*	White Stork *(Ciconia ciconia)*	4	1	3
Columbiformes	*Columbidae*	Common Woodpigeon *(Columba palumbus)*	148	69	79
		Eurasian Collared-dove *(Streptopelia decaocto)*	112	49	63
Coraciiformes	*Alcedinidae*	Eurasian Kingfisher *(Alcedo atthis)*	8	2	6
Cuculiformes	*Cuculidae*	Eurasian Cuckoo *(Cuculus canorus)*	5	3	2
Falconiformes	*Falconidae*	Peregrine Falcon *(Falco peregrinus)*	4	1	3
		Common Kestrel *(Falco tinnunculus)*	56	25	31
		Eurasian Hobby *(Falco subbuteo)*	1	1	/
Galliformes	*Phasianidae*	Common Pheasant *(Phasianus colchicus)*	5	2	3

		Red-legged Partridge<br>*(Alectoris rufa)*	1	1	/
		Common Quail<br>*(Coturnix coturnix)*	3	1	2
Gruiformes	*Rallidae*	Eurasian Coot<br>*(Fulica atra)*	2	1	1
		Common Moorhen<br>*(Gallinula chloropus)*	5	2	3
Passeriformes	*Aegithalidae*	Long-tailed Tit<br>*(Aegithalos caudatus)*	5	4	1
	Corvidae	Hooded Crow<br>*(Corvus cornix)*	155	72	83
		Eurasian Magpie<br>*(Pica pica)*	111	51	60
		Eurasian Jay<br>*(Garrulus glandarius)*	13	3	10
		Eurasian Jackdaw<br>*(Coloeus monedula)*	3	/	3
	Fringillidae	European Goldfinch<br>*(Carduelis carduelis)*	11	6	5
		Eurasian Chaffinch<br>*(Fringilla coelebs)*	24	10	14
		Hawfinch<br>*(Coccothraustes coccothraustes)*	1	/	1
		Eurasian Siskin<br>*(Spinus spinus)*	1	/	1
		European Greenfinch<br>*(Chloris chloris)*	6	2	4
		European Serin<br>*(Serinus serinus)*	11	5	6
	Hirundinidae	Common house Martin<br>*(Delichon urbicum)*	9	6	3
		Barn Swallow<br>*(Hirundo rustica)*	26	10	16
		Eurasian crag Martin<br>*(Ptyonoprogne rupestris)*	1	1	/
	Motacillidae	White Wagtail<br>*(Motacilla alba)*	5	2	3
	Muscicapidae	European pied Flycatcher<br>*(Ficedula hypoleuca)*	7	2	5
		Common Redstart<br>*(Phoenicurus phoenicurus)*	6	2	4
		Black Redstart<br>*(Phoenicurus ochruros)*	6	3	3
		European Robin<br>*(Erithacus rubecula)*	38	20	18
		Spotted Flycatcher<br>*(Muscicapa striata)*	4	/	4
	Paridae	Coal Tit<br>*(Periparus ater)*	1	1	/

		Marsh Tit *(Poecile palustris)*	1	/	1
		Great Tit *(Parus major)*	35	20	15
	Passeridae	Italian Sparrow *(Passer italiae)*	14	6	8
		Eurasian Tree Sparrow *(Passer montanus)*	2	1	1
	Phylloscopidae	Common Chiffchaff *(Phylloscopus collybita)*	2	1	1
	Regulidae	Common Firecrest *(Regulus ignicapilla)*	3	1	2
		Goldcrest *(Regulus regulus)*	17	9	8
	Sturnidae	Common Starling *(Sturnus vulgaris)*	30	18	12
	Sylviidae	Blackcap *(Sylvia atricapilla)*	14	9	5
		Dartford Warbler *(Sylvia undata)*	1	/	1
	Troglodytidae	Winter Wren *(Troglodytes troglodytes)*	1	/	1
	Turdidae	Common Blackbird *(Turdus merula)*	236	130	106
		Song Thrush *(Turdus philomelos)*	5	2	3
Pelecaniformes	*Ardeidae*	Grey Heron *(Ardea cinerea)*	18	8	10
		Cattle Egret *(Bubulcus ibis)*	14	3	11
		Little Egret *(Egretta garzetta)*	4	2	2
	Threskiornithidae	African sacred Ibis *(Threskiornis aethiopicus)*	3	3	/
Piciformes	*Picidae*	Black Woodpecker *(Dryocopus martius)*	1	1	/
		Great Spotted Woodpecker *(Dendrocopos major)*	27	13	14
		Green Woodpecker *(Picus viridis)*	43	26	17
Podicipediformes	*Podicipedidae*	Great Crested Grebe *(Podiceps cristatus)*	2	1	1
Strigiformes	*Strigidae*	Tawny Owl *(Strix aluco)*	7	3	4
		Eurasian Scops-owl *(Otus scops)*	10	5	5
		Little Owl *(Athene noctua)*	51	16	35
		Long-eared Owl *(Asio otus)*	8	1	7

	Tytonidae	Common Barn-owl<br>*(Tyto alba)*	2	1	1
Suliformes	*Phalacrocoracidae*	Great Cormorant<br>*(Phalacrocorax carbo)*	4	1	3
		Total	1654	815	839
